# Supplementary material for: Quenching effect of oscillating potential on anisotropic resonant transmission through a phosphorene electrostatic barrier
Source: Sci Rep. 2021 Feb 3;11:2881. doi: 10.1038/s41598-021-82323-z (PMC7859226; doi:10.1038/s41598-021-82323-z)
Supplement: Supplementary file 1 — Supplementary Information. [file 41598_2021_82323_MOESM1_ESM.docx]

APPENDIX:

For tunneling along the Г-Y direction, the potential profile being uniform along the x-direction one can write

$V\left( x,y,t \right)=V\left( y,t \right)= V_{0}+ V_{t}Cos\omega t$ for $0\leq y\leq d$; Region – II (A1)

= 0 elsewhere; Region – I and Region – III.

In that case the coupled differential equations for the two pseudo-spin components are given by

$\left[ \nu_{c}\frac{\partial^{2}}{\partial y^{2}}+ \rho_{c}^{m} \right] \varphi_{a}^{m}\left( y \right)=\gamma k_{x}\varphi_{b}^{m}\left( y \right)$ (A2)a

$\left[ \nu_{v}\frac{\partial^{2}}{\partial y^{2}}- \rho_{v}^{m} \right] \varphi_{b}^{m}\left( y \right)=-{\gamma k_{x}\varphi}_{a}^{m}\left( y \right)$ (A2)b

with $\rho_{c}^{m}= \left( E_{F}+m\omega-V_{0}-E_{c}-\eta_{c}k_{x}^{2} \right)$ and $\rho_{v}^{m}= \left( E_{F}+m\omega-V_{0}-E_{v}+\eta_{v}k_{x}^{2} \right)$.

The solutions for the spinor functions take the form of Eqn. (6), where $k_{i}^{m}$’s are the solutions for the qarktic equation

$f_{1}\left( k_{i}^{m} \right)^{4}+{g_{1}\left( k_{i}^{m} \right)}^{2}+h_{1}=0$ (A3)

with $f_{1}=\nu_{c}\nu_{v}$, $g_{1}=\rho_{c}\nu_{v}-\nu_{c}\rho_{v}$ and $h_{1}=\left( \gamma k_{x} \right)^{2}-\rho_{c}\rho_{v}$ .

Here $\xi_{i}^{m}=\frac{1}{h_{2}}\left[ f_{2}\left( k_{i}^{m} \right)^{2}+g_{2} \right]$, for *i* = 1, 2, 3 and 4 with $f_{2}=f_{1}$, $g_{2}=\rho_{c}\nu_{v}$ and $h_{2}=\gamma\nu_{v}k_{x}$. Further the expression for the probability current density along the x-direction corresponding to the Hamiltonian eqn. (1) is given by

$J_{y}=i\left[ \frac{\partial\psi^{ϯ}}{\partial y}N\psi-\psi^{ϯ}N\frac{\partial\psi}{\partial y} \right]$ (A4)

with the matrix $N=-2\left( \begin{matrix} \nu_{c} & 0 \\ 0 & -\nu_{v} \end{matrix} \right)$ .

Finally, the transmission coefficient is given by

$T_{m}=\frac{p_{1}^{m}\left\{ \nu_{c}-\nu_{v}\lambda_{1}^{m}{\lambda_{1}^{m}}^{*}+\beta\left( \lambda_{1}^{m}+{\lambda_{1}^{m}}^{*} \right) \right\}}{p_{1}^{0}\left\{ \nu_{c}-\nu_{v}\lambda_{1}^{0}{\lambda_{1}^{0}}^{*}+\beta\left( \lambda_{1}^{0}+{\lambda_{1}^{0}}^{*} \right) \right\}}\left| \frac{A_{3m}}{A_{10}} \right|^{2}$ (A5)
